# Dysmenorrhea and Absence of Restorative Sleep

**DOI:** 10.14740/jocmr6579

**Published:** 2026-07-31

**Authors:** Keiko Takata, Kazuhiko Kotani, Hitoshi Umino

**Affiliations:** a Department of Obstetrics and Gynecology, Uwanomachi Clinic, Kanuma, Japan; b Health Checkup Center, Utsunomiya Memorial Hospital, Utsunomiya, Japan; c Center for Community Medicine, Jichi Medical University, Shimotsuke, Japan

**Keywords:** Dysmenorrhea, Gynecological symptom, Health checkup, Pain, Sleep

## Abstract

**Background:**

Dysmenorrhea negatively affects daily functioning; however, its association with restorative sleep remains undetermined, while sleep-related health is a recent topic. This study investigated whether there is an association between dysmenorrhea severity and the absence of restorative sleep in women.

**Methods:**

This cross-sectional study included 2,752 women of 18–56 years of age who underwent health-screening. Dysmenorrhea was categorized as mild, moderate, or severe, based on self-reports. Restorative sleep was evaluated using a questionnaire regarding sufficient rest from sleep. Their associations were tested using a logistic regression analysis adjusted for confounders with odds ratio (OR) and 95% confidence interval (CI).

**Results:**

Restorative sleep was absent in 38.4% of this population. There was a significant association between severe dysmenorrhea and the absence of restorative sleep (adjusted OR 2.282, 95% CI 1.744–2.986; P < 0.001). There was also a significant association between moderate dysmenorrhea and the absence of restorative sleep (adjusted OR 1.241, 95% CI 1.049–1.467; P = 0.012).

**Conclusions:**

The results indicated a positive association between dysmenorrhea severity and the absence of restorative sleep in women. The evaluation of restorative sleep may be relevant to health checks in association with dysmenorrhea.

## Introduction

Dysmenorrhea is one of the most common conditions among women of reproductive age, and is known to impair daily functioning and be associated with a poorer quality of life [1]. Beyond pelvic pain, affected individuals frequently report fatigue, reduced concentration, and lower productivity, implying that the impact of dysmenorrhea extends beyond gynecological symptoms.

Earlier studies have demonstrated associations between menstrual symptoms and sleep disturbances, including poor sleep quality, excessive daytime sleepiness, and insomnia-related complaints. However, these studies focused mainly on sleep duration or insomnia symptoms among adolescents or young adults [2].

Recently, great attention has been paid to restorative sleep, defined as the subjective feeling of being refreshed upon awakening [3]. Restorative sleep represents a dimension distinct from insomnia and reflects physiological recovery during sleep [3]. Importantly, non-restorative sleep is associated with impaired daily functioning and adverse health outcomes [4, 5].

Despite the potential clinical importance of this concept, the association between dysmenorrhea severity and restorative sleep has not yet been explored in adult women. Therefore, the present study aimed to investigate the association between dysmenorrhea severity and the absence of restorative sleep in women.

## Materials and Methods

### Study design and participants

**Figure 1:**
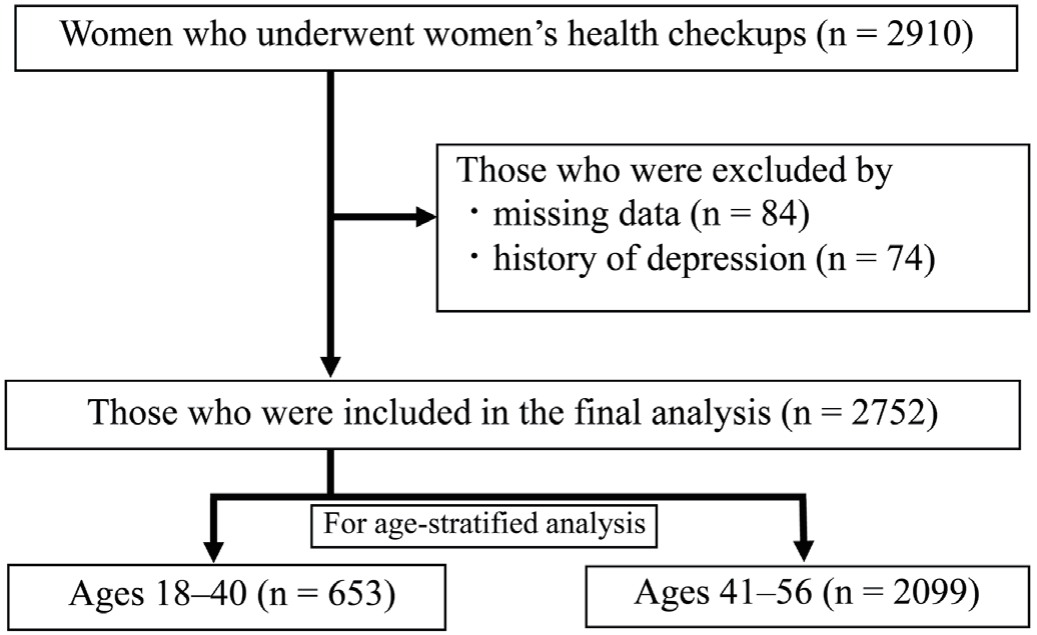
Flowchart of the participant selection process. From the initial pool of 2,910 women who underwent health checkups, participants with missing data or a history of depression were excluded. All included participants were actively menstruating women who had currently menstruated within the past year. Finally, 2,752 women were included in the analysis.

This cross-sectional study examined the data of 2,752 women of 18–56 years of age who underwent a health-screening examination and completed a self-reported questionnaire at a health checkup center where we worked. Of 2,910 women for women’s health checkups, 84 with missing data were excluded; then, those with complete data were included in the study. Furthermore, 74 with a history of depressive disorders were excluded because depressive symptoms are considered to influence sleep perception. The flowchart of selection of women analyzed is summarized in Figure 1.

Health-screening programs in Japan provide standardized physical and biochemical measurements of lifestyle-related diseases along with the assessment of lifestyle factors. Information included age, body mass index (BMI), blood low-density lipoprotein cholesterol (LDL-C) levels, fasting plasma glucose levels, current smoking habits, exercise habits (defined as participating in exercise for over 30 min per time, at least twice a week), alcohol consumption, and menstrual irregularity. We also confirmed that all women had actively menstruated within the past year since postmenopausal women could not complete the questionnaire regarding the current dysmenorrhea severity.

This study was conducted in accordance with the Declaration of Helsinki and was reviewed. It was approved in an opt-out manner by the Ethics Committee of Jichi Medical University providing participants with the opportunity to refuse the use of their data (No. 22-096).

### Evaluation of menstrual cycle and dysmenorrhea

Menstrual characteristics such as the cycle and dysmenorrhea were evaluated using self-reported questionnaire. The question was “how is your menstrual cycle pattern?”. The response options were: “regular” or “irregular.” As per the dysmenorrhea severity, the question was “how is your menstrual pain of dysmenorrhea?”. The response options were: “non/mild,” “moderate,” or “severe.”

### Evaluation of restorative sleep

Restorative sleep was also evaluated using a self-reported questionnaire. The question, as described in an earlier study [3], was “do you feel that you obtain restorative sleep?”. The response options were: “yes” or “no.” Those who answered “No” were classified as the absence of restorative sleep.

### Statistical analysis

Continuous variables are expressed as mean ± standard deviation, and categorical variables are expressed as numbers and percentages. Continuous variables were analyzed using *t*-test. Categorical variables were examined using the Chi-square test. The association between each variable and restorative sleep was examined using univariate and multivariate logistic regression models. In the multivariate model, the adjusted variables were age, BMI, LDL-C, fasting plasma glucose, current smoking, exercise, alcohol consumption, and menstrual irregularity. In particular, BMI, LDL-C, and fasting plasma glucose levels were used as covariables that are considered lifestyle-related physiological variables to potentially interact with sleep conditions. Regarding dysmenorrhea severity, women with non-to-mild symptoms were treated as a reference as those show minor symptoms in the real world. The odds ratios (ORs) and 95% confidence intervals (CIs) for the groups of dysmenorrhea with moderate and severe symptoms were calculated independently relative to the reference group. Furthermore, while considering the different clinical life stages, age-stratified analyses were performed by dividing into two groups: ages 18–40 (younger/premenopausal group) and ages 41–56 (perimenopausal group). All analyses were performed using SPSS (Ver. 28, IBM, Tokyo, Japan). Statistical significance was set at P < 0.05.

## Results

### Characteristics of the studied women

Table 1 presents the general characteristics of the studied women. The prevalence of the absence of restorative sleep was 38.4%. Women reporting the absence of restorative sleep tended to exhibit less favorable clinical and lifestyle profiles (aside from alcohol habit) as well as greater dysmenorrhea severity than those with restorative sleep.

**Table 1:** Characteristics of Studied Woman According to the Presence or Absence of Restorative Sleep

Variable	All (n = 2,752)	Restorative sleep (+) (n = 1,694)	Restorative sleep (-) (n = 1,058)	P value
Age, years	43.7 ± 4.8	43.6 ± 4.8	44.0 ± 4.8	0.016*
BMI, kg/m^2^	22.3 ± 4.0	22.1 ± 3.7	22.7 ± 4.3	< 0.001*
LDL-C, mg/dL	114 ± 29	113 ± 28	117 ± 30	< 0.001*
Fasting plasma glucose, mg/dL	95 ± 10	95 ± 11	95 ± 10	0.498
Current smoking habit, n (%)	262 (9.5%)	154 (9.1%)	108 (10.2%)	0.331
Exercise habit, n (%)	364 (13.2%)	248 (14.6%)	116 (11.0%)	0.006*
Alcohol habit, n (%)	1,338 (48.6%)	870 (51.4%)	468 (44.2%)	< 0.001*
Menstrual irregularity, present	673 (24.5%)	367 (21.7%)	306 (28.9%)	< 0.001*
Dysmenorrhea				< 0.001*
Non-to-mild state	1,377 (50.0%)	898 (53.0%)	479 (45.3%)	
Moderate state	1,102 (40.0%)	671 (39.6%)	431 (40.7%)	
Severe state	273 (10.0%)	125 (7.4%)	148 (14.0%)	

Data are shown as the mean ± standard deviation, median (interquartile range), and percentage. P-values represent the difference in each variable among women with mild to severe dysmenorrhea (by Chi-square test or analysis of variance). *P < 0.05, in comparison to the mild dysmenorrhea group (by residual tests following Chi-squared test or *t*-test). BMI: body mass index; LDL-C: low-density lipoprotein cholesterol.

### Association between dysmenorrhea severity and absence of restorative sleep

As shown in Table 2, in the univariate analysis, there was a significant association between severe dysmenorrhea and the absence of restorative sleep. In the multivariate analysis, after adjustment for all confounders, the association remained significant (adjusted OR 2.282, 95% CI 1.744–2.986; P < 0.001). There was also a significant association between moderate dysmenorrhea and the absence of restorative sleep, independent of all confounders (adjusted OR 1.241, 95% CI 1.049–1.467; P = 0.012). The OR increased stepwise in a dose–response manner with dysmenorrhea severity.

**Table 2:** Association of Each Variable With Absence of Restorative Sleep

Variable	Univariate analysis		Multivariate analysis	
	OR (95% CI)	P value	OR (95% CI)	P value
Age, years	1.020 (1.004–1.037)	0.016*	1.017 (1.000–1.035)	0.057
BMI, kg/m^2^	1.037 (1.017–1.057)	< 0.001*	1.026 (1.005–1.048)	0.017*
LDL–C, mg/dL	1.005 (1.002–1.007)	< 0.001*	1.002 (1.000–1.005)	0.113
Fasting plasma glucose, mg/dL	1.003 (0.995–1.010)	0.500	1.000 (1.000–1.006)	0.547
Current smoking habit, presence	1.137 (0.877–1.473)	0.332	1.074 (0.823–1.400)	0.600
Exercise habit, absence	1.393 (1.101–1.762)	0.006*	1.409 (1.108–1.792)	0.005*
Alcohol habit, presence	0.751 (0.644–0.877)	< 0.0001*	0.767 (0.654–0.898)	0.001*
Menstrual irregularity, presence	1.471 (1.234–1.755)	< 0.001*	1.368 (1.138–1.644)	0.001*
Dysmenorrhea: moderate vs. non-to-mild state	1.048 (0.896–1.226)	0.557	1.241 (1.049–1.467)	0.012*
Dysmenorrhea: severe vs. non-to-mild state	2.041 (1.587–2.626)	< 0.001*	2.282 (1.744–2.986)	< 0.001*

*P < 0.05. BMI: body mass index; CI: confidence interval; LDL-C: low-density lipoprotein cholesterol; OR: odds ratio.

### Association between absence of restorative sleep and other variables

In addition, in the univariate analysis, there was a significant association between the higher BMI level and the absence of restorative sleep. There was also a significant association between the presence of alcohol consumption and the absence of restorative sleep. Furthermore, there was a significant association between the menstrual irregularity and the absence of restorative sleep. In the multivariate logistic regression analysis, there remained to be a significant independent association between these variables and the absence of restorative sleep.

### Age-stratified analyses

The two groups of ages 18–40 (n = 653) and ages 41–56 (n = 2,099) were analyzed, respectively. In the former younger/premenopausal group, the prevalence of the absence of restorative sleep was 35.1% (Table 3). Women reporting the absence of restorative sleep tended to exhibit less favorable clinical and lifestyle profiles as well as greater dysmenorrhea severity than those with restorative sleep. As shown in Table 4, there was a significant independent association between severe dysmenorrhea and the absence of restorative sleep in the multivariate analysis (adjusted OR 2.569, 95% CI 1.545–4.272; P < 0.001). In the latter perimenopausal group, the prevalence of the absence of restorative sleep was 39.5% (Table 5). Women reporting the absence of restorative sleep tended to exhibit less favorable clinical and lifestyle profiles (aside from alcohol habit) as well as greater dysmenorrhea severity than those with restorative sleep. As shown in Table 6, in the multivariate analysis, there was a significant independent association between moderate dysmenorrhea and the absence of restorative sleep (adjusted OR 1.219, 95% CI 1.009–1.474; P = 0.040) as well as between severe dysmenorrhea and the absence of restorative sleep (adjusted OR 2.242, 95% CI 1.624–3.095; P < 0.001).

**Table 3:** Characteristics of Studied Woman Aged 18–40 According to the Presence or Absence of Restorative Sleep

Variable	All (n = 653)	Restorative sleep (+) (n = 424)	Restorative sleep (-) (n = 229)	P value
Age, years	37.1 ± 2.8	37.2 ± 2.8	37.0 ± 3.0	0.456
BMI, kg/m^2^	21.8 ± 4.1	21.5 ± 3.8	22.2 ± 4.7	0.038*
LDL-C, mg/dL	106 ± 28	104 ± 27	109 ± 31	0.057
Fasting plasma glucose, mg/dL	93 ± 9	93 ± 7	93 ± 12	0.590
Current smoking habit, n (%)	61 (9.3%)	41 (9.7%)	20 (8.7%)	0.695
Exercise habit, n (%)	66 (10.1%)	50 (11.8%)	16 (7.0%)	0.052
Alcohol habit, n (%)	322 (49.3%)	221 (52.1%)	101 (44.1%)	0.051
Menstrual irregularity, present	112 (17.2%)	57 (13.4%)	55 (24.0%)	< 0.001*
Dysmenorrhea				< 0.001*
Non-to-mild state	246 (38.0%)	175 (41.3%)	73 (31.9%)	
Moderate state	313 (47.9%)	203 (47.9%)	110 (48.0%)	
Severe state	92 (14.1%)	46 (10.8%)	46 (20.1%)	

Data are shown as the mean ± standard deviation, median (interquartile range), and percentage. P-values represent the difference in each variable among women with mild to severe dysmenorrhea (by chi-square test or analysis of variance). *P < 0.05, in comparison to the mild dysmenorrhea group (by residual tests following Chi-squared test or *t*-test). BMI, body mass index; LDL-C, low-density lipoprotein cholesterol.

**Table 4:** Association of Each Variable With Restorative Sleep in Studied Woman Aged 18–40

Variable	Univariate analysis		Multivariate analysis	
	OR (95% CI)	P value	OR (95% CI)	P value
Age, years	0.979 (0.925–1.035)	0.454	0.982 (0.926–1.041)	0.537
BMI, kg/m^2^	1.041 (1.002–1.082)	0.040*	1.036 (0.992–1.083)	0.111
LDL-C, mg/dL	1.005 (1.000–1.011)	0.058	1.003 (0.997–1.009)	0.391
Fasting plasma glucose, mg/dL	1.005 (0.988–1.022)	0.591	0.997 (0.978–1.016)	0.732
Current smoking habit, presence	0.894 (0.510–1.566)	0.695	0.751 (0.415–1.361)	0.345
Exercise habit, absence	1.780 (0.989–3.203)	0.054	1.889 (1.022–3.492)	0.042*
Alcohol habit, presence	0.725 (0.525–1.001)	0.051	0.738 (0.527–1.035)	0.078
Menstrual irregularity, presence	2.035 (1.348–3.073)	< 0.001*	1.914 (1.246–2.939)	0.003*
Dysmenorrhea: moderate vs. non-to-mild state	1.006 (0.729–1.388)	0.969	1.360 (0.942–1.965)	0.101
Dysmenorrhea: severe vs. non-to-mild state	2.066 (1.323–3.224)	0.001*	2.569 (1.545–4.272)	< 0.001*

*P < 0.05. BMI: body mass index; CI: confidence interval; LDL-C: low-density lipoprotein cholesterol; OR: odds ratio.

**Table 5:** Characteristics of Studied Woman Aged 41-56 According to the Presence or Absence of Restorative Sleep

Variable	All (n = 2,099)	Restorative sleep (+) (n = 1,270)	Restorative sleep (-) (n = 829)	P value
Age, years	45.8 ± 3.1	45.7 ± 3.1	45.9 ± 3.1	0.066
BMI, kg/m^2^	22.5 ± 3.9	22.3 ± 3.7	22.8 ± 4.2	0.003*
LDL-C, mg/dL	117 ± 29	115 ± 28	119 ± 29	0.008*
Fasting plasma glucose, mg/dL	95 ± 11	95 ± 11	95 ± 10	0.767
Current smoking habit, n (%)	201 (9.6%)	113 (8.9%)	88 (10.6%)	0.191
Exercise habit, n (%)	298 (14.2%)	198 (15.6%)	100 (12.1%)	0.024*
Alcohol habit, n (%)	1,016 (48.4%)	649 (51.1%)	367 (44.3%)	0.002*
Menstrual irregularity, present	561 (26.7%)	310 (24.4%)	251 (30.2%)	0.003*
Dysmenorrhea				< 0.001*
Non-to-mild state	1,129 (53.8%)	723 (56.9%)	406 (49.0%)	
Moderate state	789 (37.6%)	468 (36.9%)	321 (38.7%)	
Severe state	181 (8.6%)	79 (6.2%)	102 (12.3%)	

Data are shown as the mean ± standard deviation, median (interquartile range), and percentage. P-values represent the difference in each variable among women with mild to severe dysmenorrhea (by chi-square test or analysis of variance). *P < 0.05, in comparison to the mild dysmenorrhea group (by residual tests following Chi-squared test or *t-*test). BMI: body mass index; LDL-C: low-density lipoprotein cholesterol.

**Table 6:** Association of Each Variable With Restorative Sleep in Studied Woman Aged 41–56

Variable	Univariate analysis		Multivariate analysis	
	OR (95% CI)	P value	OR (95% CI)	P value
Age, years	1.027 (0.998–1.056)	0.066	1.019 (0.988–1.051)	0.232
BMI, kg/m^2^	1.034 (1.011–1.057)	0.003*	1.024 (0.999–1.050)	0.056
LDL-C, mg/dL	1.004 (1.001–1.007)	0.009*	1.002 (0.999–1.005)	0.208
Fasting plasma glucose, mg/dL	1.001 (0.993–1.009)	0.767	0.997 (0.988–1.007)	0.583
Current smoking habit, presence	1.216 (0.907–1.631)	0.192	1.179 (0.873–1.591)	0.282
Exercise habit, absence	1.346 (1.040–1.743)	0.024*	1.335 (1.027–1.736)	0.031*
Alcohol habit, presence	0.760 (0.638–0.906)	0.002*	0.773 (0.646–0.925)	0.005*
Menstrual irregularity, presence	1.345 (1.106–1.636)	0.003*	1.256 (1.020–1.546)	0.032*
Dysmenorrhea				
Moderate vs. non-to-mild state	1.083 (0.904–1.297)	0.387	1.219 (1.009–1.474)	0.040*
Dysmenorrhea: severe vs. non-to-mild state	2.115 (1.555–2.878)	< 0.001*	2.242 (1.624–3.095)	< 0.001*

*P < 0.05. BMI: body mass index; CI: confidence interval; LDL-C: low-density lipoprotein cholesterol; OR: odds ratio.

## Discussion

The present study revealed a significant independent association between dysmenorrhea severity and the absence of restorative sleep in women. The results indicate that the evaluation of restorative sleep may be relevant to health checks in association with dysmenorrhea, considering the importance of restorative sleep in daily life and health conditions [4, 5]. This should stimulate further studies on restorative sleep in adult women.

There are several possible explanations for this finding. In general, dysmenorrhea disrupts sleep continuity. Uterine contractions mediated by prostaglandins generate intermittent nociceptive inputs, which may increase nocturnal arousal and micro-awakening [1]. This mechanism may impair sleep depth and can lead to a reduced sense of recovery upon awakening.

Pain and sleep are further known to have a bidirectional relationship [6]. While menstrual pain can disrupt sleep, reduced sleep recovery may impair the descending pain inhibitory pathways and inflammation-pain regulation, thereby lowering pain thresholds [7]. Sleep disturbances may also impair endogenous pain inhibitory systems [8, 9]. These are deemed to explain the complex association between dysmenorrhea severity and the absence of restorative sleep.

The present study observed the dose–response association of dysmenorrhea severity with the absence of restorative sleep, which is of interest. Given the bidirectional relationship between pain and sleep [7–9], worsening dysmenorrhea and poorer sleep functions are presumed to exacerbate synergistically both conditions. The mechanistical explanations of this observation would be required.

Restorative sleep represents a dimension distinct from insomnia symptoms and is also involved in daytime functioning [3]. Individuals with non-restorative sleep exhibit fatigue, reduced activity, and biological alterations [4]. Thus, it is of note that dysmenorrhea may influence the “overall” functional health in association with sleep recovery.

Bearing such explanations in mind, it is expected that the mechanisms of the findings and effective strategies against restorative sleep will be clarified. In addition, the present study’s findings are useful in directing future research to elucidate a potential pathway linking gynecological symptoms to broader health consequences, using the restorative sleep adopted in the present study as a clue.

In the present study, there was the significant independent association of the higher BMI level and the presence of alcohol consumption with the absence of restorative sleep. An earlier study demonstrated the association of unfavorable lifestyle factors, including obesity and alcohol consumption, with non-restorative sleep [10]. The patients with obesity are thought to have an impaired sleep architecture by a low-grade systemic inflammation, metabolic dysregulation, and sleep-disordered breathing, all of which can reduce subjective sleep recovery [10]. Alcohol consumption, although it is perceived as a sleep inducer in some cases, fragments sleep structure and suppress rapid eye movement sleep, thereby reducing restorative sleep quality [10]. The present study appears to support the findings of earlier study [10].

The present study showed that there was a significant independent association of menstrual irregularity with the absence of restorative sleep. Biological circadian rhythm and sleep patterns are known to be closely linked to neuroendocrine regulation [11]. Disruption of circadian clock gene expression can alter gonadotropin-releasing hormone pulsatility and ovarian steroidogenesis, potentially leading to menstrual irregularity. Conversely, hormonal instability associated with irregular cycles can influence sleep regulation. Thus, the association between menstrual irregularity and the absence of restorative sleep may be partly explained by the etiology underlying neuroendocrine dysregulation.

It is important to notice that the clinical features of dysmenorrhea can differ across life stages. First, primary dysmenorrhea is typically mild and/or highly prevalent among adolescents and young adults. Second, secondary dysmenorrhea, associated with conditions like endometriosis or adenomyosis in premenopausal women, involves chronic pelvic pain and inflammation that frequently cause middle-of-the-night awakenings. Third, mild menstrual cramps re-occur during perimenopause, intersecting with hormonal fluctuations. Although the association between dysmenorrhea and restorative sleep across life stages is of concern, age-stratified analysis in the present study did not reveal large difference in the association between severe dysmenorrhea and the absence of restorative sleep (their associations also appeared to be similar with those of the analysis of all ages). Multifaceted analyses will be warranted to confirm it.

The present study has several limitations. First, this is based on the cross-sectional design, which precludes any inference of causality. Second, the study was conducted in a health-screening setting. Thus, the generalizability of the findings should be careful. Third, dysmenorrhea severity was evaluated using not quantitative measures such as a visual pain score but a self-reported qualitative scale (from none/mild to severe), while this approach shows feasibility and simplicity in the health-screening setting. Fourth, because the study was conducted in health checkups, the available data were limited. The data on specific conditions, such as pregnancy, and pathologies, such as anorexia nervosa, psychiatric disorders, or endocrine disorders, could not be applied to the study, while it is unlikely that they would select health checkups if treatment in clinics takes precedence. Additional information on comorbidities such as the presence of diabetes and hypertension, as well as medication uses were not collected. We may also require the unmeasured confounding factors, including the history of gynecological surgeries, the use of hormonal contraceptives or intrauterine devices, and the frequency or type of analgesic use for dysmenorrhea, which could influence both dysmenorrhea and sleep. To classify dysmenorrhea into primary, secondary, or perimenopausal types, although we can further require the data on the presence of endometriosis or adenomyosis, parity, and vasomotor symptoms (e.g., night sweats or hot flashes), these were unavailable. The present study findings should be interpreted with caution. Finally, we did not collect the data on the duration of dysmenorrhea or sleep disorders (e.g., months and years). Since chronic symptoms are associated with lower quality of life and therapeutic strategies, tracking the clinical duration of such conditions can be a critical issue. We plan to address these limitations in future studies.

In conclusion, the results of the present study indicate a significant independent association between dysmenorrhea severity and the absence of restorative sleep in women. The evaluation of restorative sleep may be relevant to health checks in association with dysmenorrhea. Further studies are required to address this topic.

## Data Availability

The data are available from the corresponding author upon reasonable request.

## References

[1] Iacovides S, Avidon I, Baker FC (2015). What we know about primary dysmenorrhea today: a critical review. Hum Reprod Update.

[2] Jeon B, Baek J (2023). Menstrual disturbances and its association with sleep disturbances: a systematic review. BMC Womens Health.

[3] Riemann D (2013). Nonrestorative sleep: a new perspective. Sleep.

[4] Zhang J, Lamers F, Hickie IB, He JP, Feig E, Merikangas KR (2013). Differentiating nonrestorative sleep from nocturnal insomnia symptoms: demographic, clinical, inflammatory, and functional correlates. Sleep.

[5] Yoshiike T, Utsumi T, Matsui K, Nagao K, Saitoh K, Otsuki R, Aritake-Okada S (2022). Mortality associated with nonrestorative short sleep or nonrestorative long time-in-bed in middle-aged and older adults. Sci Rep.

[6] Finan PH, Goodin BR, Smith MT (2013). The association of sleep and pain: an update and a path forward. J Pain.

[7] Haack M, Simpson N, Sethna N, Kaur S, Mullington J (2020). Sleep deficiency and chronic pain: potential underlying mechanisms and clinical implications. Neuropsychopharmacology.

[8] Herrero Babiloni A, Brazeau D, Jodoin M, Theis-Mahon N, Martel MO, Lavigne GJ, Moana-Filho EJ (2024). The impact of sleep disturbances on endogenous pain modulation: a systematic review and meta-analysis. J Pain.

[9] Ishikura IA, Hachul H, Tufik S, Andersen ML (2023). Dysmenorrhea and sleep: a review. Sleep Med Clin.

[10] Wakasugi M, Kazama JJ, Narita I, Iseki K, Moriyama T, Yamagata K, Fujimoto S (2014). Association between combined lifestyle factors and non-restorative sleep in Japan: a cross-sectional study based on a Japanese health database. PLoS One.

[11] Beroukhim G, Esencan E, Seifer DB (2022). Impact of sleep patterns upon female neuroendocrinology and reproductive outcomes: a comprehensive review. Reprod Biol Endocrinol.

